# Higher patient satisfaction with antidepressants correlates with earlier drug release dates across online user‐generated medical databases

**DOI:** 10.1002/prp2.355

**Published:** 2017-09-04

**Authors:** Scott Siskind, Roland C. Aydin, Punit Matta, Christian J. Cyron

**Affiliations:** ^1^ St. Mary Mercy Hospital Saint Joseph Mercy Health System Livonia Michigan; ^2^ Technische Universität München Institute for Computational Mechanics Garching Germany; ^3^ Tufts University Medford Massachusetts

**Keywords:** Clinical Pharmacology, disease management, patient‐generated online data, Pharmacometrics, Psychopharmacology

## Abstract

Studies establishing the use of new antidepressants often rely simply on proving efficacy of a new compound, comparing against placebo and single compound. The advent of large online databases in which patients themselves rate drugs allows for a new Big Data–driven approach to compare the efficacy and patient satisfaction with sample sizes exceeding previous studies. Exemplifying this approach with antidepressants, we show that patient satisfaction with a drug anticorrelates with its release date with high significance, across different online user‐driven databases. This finding suggests that a systematic reevaluation of current, often patent‐protected drugs compared to their older predecessors may be helpful, especially given that the efficacy of newer agents relative to older classes of antidepressants such as monoamine oxidase inhibitors (MAOIs) and tricyclic antidepressants (TCAs) is as yet quantitatively unexplored.

**Abbreviations**
MAOIs: monoamine oxidase inhibitors
TCAs: tricyclic antidepressants

## Introduction

Direct comparisons between medications are commonly expensive and time‐consuming endeavors, even though they are of significant economic importance (Zentner et al. 2005; Clement et al. 2009). Currently, the FDA approval process for new medication focuses on efficacy over placebo, or at best compared to a selected previous treatment (Pande et al. 1996), rather than noninferiority to the whole set of previously established treatments (Hanrahan and New 2014). Even using such an uncomplicated criterion, the data gathering of the phase II and phase III trials that establish safety and efficacy remains the most expensive bottleneck in the drug development pipeline (Sertkaya et al. 2016). Given the high economic incentives of generating new and approved compounds as older medications lose their patent protection and thus their profitability, the interest in financing further high‐powered studies comparing newer‐ to older‐generation medications is often limited. There have been some prior comparative studies between different generations of antidepressants, but their intrinsic limitation to comparatively few data points and often only pairwise comparisons have limited their influence on overall market shares and treatment choices (Cipriani et al. 2009).

The dichotomy between expensive comparative studies that the pharmaceutical industry has little incentive to conduct, and medical anecdotes and single case studies which abound but are of limited generalizability and thus have a low degree of external validity has so far precluded an effective and affordable approach to drug comparisons. The dearth of comparative drug data has been tentatively alleviated through the advent of meta‐analyses which aggregate smaller studies in order to increase the predictive power along with the sample size of the dataset. Such meta‐analyses have been deployed for antidepressants in particular (Anderson 2000; Gorman et al. 2002; Thase et al. 2006; Cipriani et al.).

However, in order to benefit from the big data paradigm that recently has proved fruitful for optimizing fields ranging from retail to systems management to software development, an alternative source of data is needed (Gandomi and Haider 2015).

A big data‐based approach could enable a prevetting of which substances are good candidates to yield positive results in comparative trials, relying on evidence‐based standards rather than anecdotal data points (Hughes and Cohen 2011).

In this study, we demonstrate that large online databases can be used to perform low‐cost comparisons between a large number of different drugs. A first result of the application of this novel paradigm to the examination of the efficacy of antidepressants is that the drug release date strongly negatively correlates with user satisfaction both for the self‐reported indications of depression and major depression, such that older drugs have on average significantly higher scores than newer drugs throughout the major online databases used in this study. The choice of the indications depression and major depression was taken as due to the nature of these conditions, patient satisfaction constitutes a particularly close proxy to effectiveness of care (Linn and Greenfield 1982; Hansson et al. 1994; Wyshak and Barsky 1994, 1995). For this field, as with many others, there is a relative dearth of studies comparing older classes of antidepressants such as monoamine oxidase inhibitors (MAOIs) and tricyclic antidepressants (TCAs) (Stewart and Thase 2007; Cohen et al. 2012; Goldberg and Thase 2013).

Different explanations for this finding have to be taken into consideration, each with important therapeutic and policy implications. While this study cannot conclusively answer the causation of this relationship, it uses the advent of a large reservoir of online user‐generated data to raise an important question: Are new drugs (that are typically patent protected and thus more expensive) superior to older drugs or may actually even the opposite be true?

## Materials and Methods

### Data acquisition

Eligible databases were required to satisfy several criteria in order to be included in this study.

First, they needed to provide a sample size sufficient for representing the spectrum of experiences. This concern can be addressed by choosing only among the largest public health databases. While there is no generic answer on the minimum size of a dataset in order to usefully apply data mining, the larger the sample the more reliably and with higher fidelity actual relationships will be represented in the data (Halevy et al. 2009; Rajaraman 2011).

Second, information on the demographic structure of users should ideally be available. A major caveat with using preexisting large collections of data is that the respondents are not independent and identically distributed to the patient base as a whole; the structure of the sample is skewed by selecting only those patients both versed in current technology and willing to leave feedback. Over time, technological literacy as a bias in selection loses its salience as online competence increases and use of public platforms becomes more ubiquitous. Generally, reporting bias manifests itself such that unsolicited feedback tends to gravitate toward extreme outcomes, as those cause stronger motivational incentives to leave public feedback (The Sound of Silence in Online Feedback, 2007). However, as a comparison relies on the relative performance of drugs and a reporting bias would apply across the spectrum of drugs, the relationships between different drugs as translated through the prism of reporting bias maintain their ordering, as can be verified when comparing data points to previously done comparative studies such as Cipriani et al.

Third, the patient feedback needed not only to be specific to the medication, but also specific to an indication as well. The study's internal validity could be compromised by intermingling patient feedback given for one indication with feedback given for another indication treated with the same drug, especially as many medications have various and differing off‐label uses (Table 1).

**Table 1:** Rankings for the indications of depression and major depression based on weighted averages for all generics and brand names using that active compound, over all patient‐populated databases used in this study

Generic ranking (year of earliest FDA approval)	Depression‐weighted average score	Generic ranking (year of earliest FDA approval)	Major depression‐weighted average score
Niacin (1957)	9.60	Imipramine (1959)	9.70
Tramadol (1995)	9.30	Tranylcypromine (1961)	8.40
Amoxapine (1980)	9.17	Modafinil (1998)	8.00
Fluoxetine + Olanzapine (2003)	8.99	Phenelzine (1961)	8.00
Alprazolam (1981)	8.61	Fluoxetine (1987)	7.26
Lamotrigine (1994)	8.40	Sertraline (1991)	7.19
Clomipramine (1989)	8.37	Lamotrigine (1994)	7.15
Nefazodone (1994)	8.30	Selegiline (1989)	7.10
Selegiline (1989)	8.22	Fluoxetine + Olanzapine (2003)	7.03
Modafinil (1998)	8.20	Escitalopram (2002)	7.01
Tranylcypromine (1961)	8.08	Bupropion (1985)	6.94
Phenelzine (1961)	8.05	Fluvoxamine (1994)	6.80
Isocarboxazid (1959)	8.00	Brexpiprazole (2015)	6.77
Methylphenidate (1955)	7.98	Venlafaxine (1993)	6.76
L‐Methylfolate	7.87	Nefazodone (1994)	6.76
Armodafinil (2007)	7.50	Trazodone (1981)	6.69
Imipramine (1959)	7.19	Olanzapine (1996)	6.67
Brexpiprazole (2015)	7.17	Risperidone (1993)	6.66
Risperidone (1993)	7.00	Mirtazapine (1996)	6.64
Maprotiline (1980)	6.99	Aripiprazole (2002)	6.43
Desipramine (1964)	6.96	Desvenlafaxine (2008)	6.32
Citalopram (1998)	6.91	Vortioxetine (2013)	6.24
Escitalopram (2002)	6.91	Duloxetine (2004)	6.21
Fluoxetine (1987)	6.83	Paroxetine (1992)	6.01
Trazodone (1981)	6.81	Quetiapine (1997)	5.62
Lithium (1970)	6.80	Vilazodone (2011)	5.48
Vilazodone (2011)	6.80	Citalopram (1998)	5.27
Duloxetine (2004)	6.70	Levomilnacipran (2013)	4.95
Maprotiline (1980)	6.67		
Bupropion (1985)	6.65		
Amitriptyline (1961)	6.58		
Trimipramine (1979)	6.57		
Fluvoxamine (1994)	6.51		
Mirtazapine (1996)	6.38		
Venlafaxine (1993)	6.37		
Nortriptyline (1964)	6.24		
Vortioxetine (2013)	6.15		
Aripiprazole (2002)	6.10		
Doxepin (1969)	6.03		
Quetiapine (1997)	5.97		
Olanzapine (1996)	5.70		
Paroxetine (1992)	5.12		
Levomilnacipran (2013)	4.89		

Only compounds with a score based on at least five reviews were considered. The average sample size for depression is 414 per compound (median 72, range 6‐2827), the average sample size for major depression is 201 per compound (median 83, range 5‐2049). Combined rankings for depression and major depression**.**

Fourth, some of the prominent mechanisms that may invalidate patient feedback need to be addressed. Drugs with an increased addictive potency may lead to subjective feedback that does not adequately reflect that medication's therapeutic effectiveness, but merely its addictive potency. Also, for nonpsychiatric indications effectiveness is often measured using surrogate markers such as serum levels of LDL for cholesterol‐lowering agents, not the patient's subjective perception. For the chosen indications of depression and major depression, there is a more immediate relationship between the diagnostic criteria as outlined in ICD‐10 (Organization WH, 1992), and symptoms as experienced by the patient.

The databases WebMD, Drugs.com, AskAPatient, and RateRx best addressed the majority of requirements previously outlined, and were thus included for this study. This choice may not be overly constrained, as the robustness of the results indicates that they may transfer to other databases. As outlined, choosing only antidepressants avoided addictive potency as a relevant confounder, and also allowed to take the subjective efficacy as a surrogate parameter closer to its objective efficacy than is the case for other, nonpsychiatric indications.

WebMD is a New York–based online publisher of health‐related information founded in 1996. It is regarded as a leading health publisher in the United States, as measured by the number of unique users per month. For the public ratings database, the questions posed were “Did the medication work for you?”, “Was it easy for you to use?”, and “Were you overall satisfied?”. Ratings for each category vary between 1 (very unhappy) and 5 (very satisfied). This study used the overall patient satisfaction rating as the basis of comparison with the other patient databases. Ratings are given only in conjunction with the indication for which the drug was prescribed.

AskAPatient was founded in 2000 as an independent website operated by the Virginia‐based Consumer Health Resource Group, LLC. It focuses on providing a public forum as a resource for drug ratings, and is likewise meant for use by healthcare consumers directly, without any affiliations with the pharmaceutical industry. The drug ratings for AskAPatient are on a scale of 1 (“dissatisfied”) to 5 (“very satisfied”), corresponding to WebMD's format. It also allows for the specific indication for the drug use to be added.

The Drugs.com. website was officially launched in September 2001 and is currently owned and operated by the privately held New Zealand–based “Drugsite Trust”. User reviews are on a scale of 1 (corresponding to “not effective”) to 10 (“most effective”). The guidelines accompanying that scale explain that notion of effectiveness to be interpreted as how positive of an experience one had with the drug, allowing for a reasonable comparison with the “satisfaction” criterion of WebMD and AskAPatient. Ratings for Drugs.com are also specific to an indication, with the overall average rating being the average over the ratings for all conditions.

RateRX has been launched in 2015, and provides a database for use by U.S. doctors to provide a large‐scale evaluation of medications for treatments”. Due to its restriction to medical doctors, the number of ratings is an order of magnitude below the patient‐populated databases. Ratings there are specific for an indication, and from a scale of 1 to 5. For the purposes of this study, it has only been used to provide a preliminary observation provided in the Discussion section. It is owned by HealthTap,

All of the aforementioned databases only remove ratings for reasons of profanity, personally identifying information, copyright violations, or similar violations of their respective terms and conditions. RateRx is an exception, as doctors there have the option to have their rating be personally identified. This analysis is only incorporating ratings based on a minimum of five samples. Different formulations of the same pharmacological agent (e.g., different brands) are grouped under the year for which the active agent was first approved. To identify similarities and differences in the characteristics, the linear regression approach of calculating Pearson correlation coefficients was chosen. Calculations are only applied for a minimum of 10 or more, and 20 or more samples per compound, in order to confirm that the results are not based on spurious correlations and on the choice of minimum reviews required for inclusion in the analysis.

It is not currently possible to determine if the same patient has given more than one response, either providing multiple ratings for one drug over time, or providing ratings for several drugs.

For the indication of depression, the weighted average between the databases is derived of an average sample size of 230 reviews per compound when considering all drugs using that compound with five or more reviews (Table 1), 267 reviews when considering drugs with 10 or more reviews, and 353 reviews when considering drugs with 20 or more reviews.

For the indication of major depression, the respective number of reviews of which the weighted average is based amounted to 108 per drug when considering all drugs using that compound with five or more reviews (Table 1), 138 reviews when considering drugs with 10 or more reviews, and 165 reviews when considering drugs with 20 or more reviews.

Lastly, for all indications, the average number of reviews per included drug was 824 per drug when considering all drugs using that compound with five or more reviews, 894 reviews when considering drugs with 10 or more reviews, and 1018 reviews when considering drugs with 20 or more reviews.

## Results

The correlation of newer drugs with a lower patient satisfaction, or conversely of older drugs with a higher patient satisfaction, was found to be the case near universally, both for the patient‐reported indication of depression, for the patient‐reported indication for major depression, as well as for all indications.

**Figure 1:**
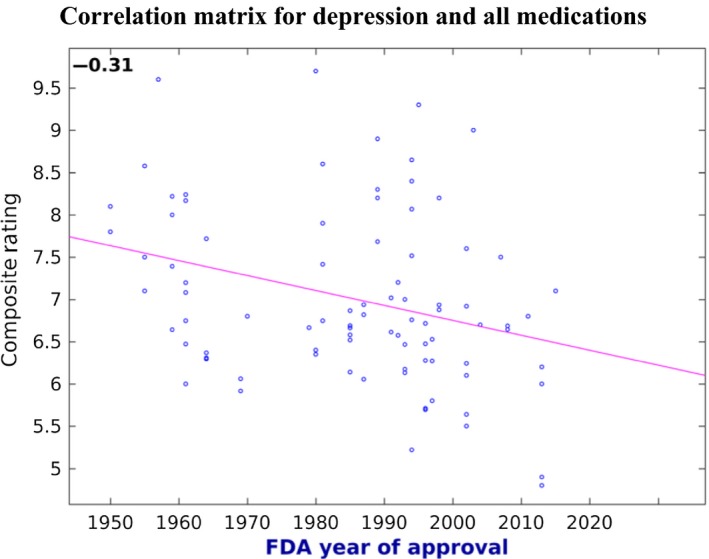
Correlation between the FDA approval date and the weighted averages for all generics and brand names rated for the indication of depression, over all patient‐populated databases used in this study. Only compounds with a score based on at least five reviews were considered. Pearson's *r* = −0.31, *P* = 0.0037. Least square regression indicated by the red boundary.

For depression the correlation held true unequivocally whether considering drugs with at least five reviews (*P* = 0.0037), at least 10 reviews (*P* = 0.0073), or at least 20 reviews (*P* = 0.0170) (Table 2). Out of a rating of 0 to 10, the weighted rating across the patient‐generated databases considered was on average 0.2 higher per decade since the FDA approval, corresponding to a Pearson's *R* of −0.31 (Fig. 1). With 95% of the composite ratings within a range of 3.9 points (out of 10), this corresponds to an average change of 5.1 percentile points per decade within that range.

**Table 2:** Correlations between year of FDA approval and weighted average rating for all separate brands with more than the indicated number of samples across the online databases in this study, using the date of FDA approval for that brand (not the earliest FDA approval for the active compound)

Number of samples required for inclusion	Depression		Major depression		All indications	
	Pearson's *r*	*P*‐value	Pearson's *r*	*P*‐value	Pearson's *r*	*P*‐value
≥ 1	−0.07	0.4680	−0.05	0.6574	−0.2	**0.0282**
≥ 5	−0.30	**0.0037**	−0.56	**<0.0001**	−0.2	**0.0329**
≥ 10	−0.30	**0.0073**	−0.60	**0.0001**	−0.18	0.0721
≥ 20	−0.31	**0.0170**	−0.60	**0.0002**	−0.24	**0.0224**

Shown are the Pearson correlation coefficient r (denoting the strength of the correlation between the rating and the year of FDA approval of a drug), and the associated *P*‐value. The Bonferroni‐adjusted *P*‐value for significance is P* = 0.017. All brands: correlation between year of FDA approval and weighted average rating. Values smaller than the (unadjusted) P‐value of 0.05 are denoted in bold.

**Figure 2:**
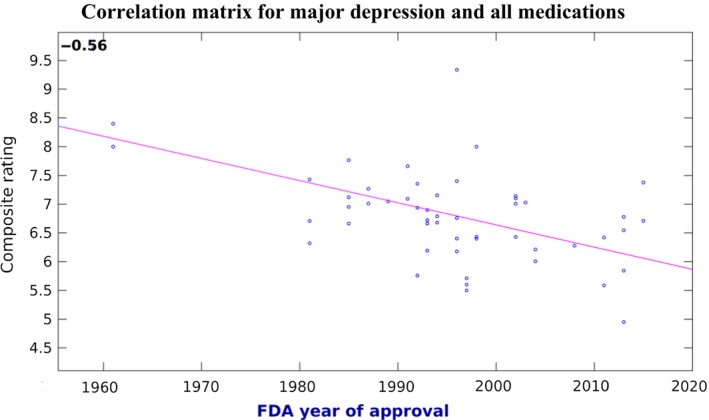
Correlation between the FDA approval date and the weighted averages for all generics and brand names rated for the indication of major depression, over all patient‐populated databases used in this study. Only compounds with a score based on at least five reviews were considered. Pearson's *r* = −0.56, *P* ≤ 0.0001. Least square regression indicated by the red boundary.

For major depression, the same relationship was even more pronounced, with an average loss of 0.4 rating points out of 10 per decade since the FDA approval (corresponding to a Pearson's *R* of between −0.56), highly significant for all drugs with at least five reviews (*P* ≤ 0.0001), at least 10 reviews (*P* ≤ 0.0001), or at least 20 reviews (*P* = 0.0002) (Table 2). With 95% of the composite ratings within a range of 3.6 points (out of 10), this corresponds to an average change of 11.1 percentile points per decade within that range (Fig. 2).

When reviews for the various indications other than depression or major depression are included, it becomes evident that the correlation remains, albeit in a weaker manifestation that passes the 0.05 threshold but not the conservatively adjusted P* value for multiple comparisons. While differing thresholds for the required number of reviews per drug do not constitute independent hypotheses on the same dependent variable, the tests for different indications (depression, major depression, all indications) are in this study conservatively considered as independent hypotheses. In order to correct for multiple hypothesis testing using Bonferroni correction, an adjusted *P*‐value for statistical significance is given by P* = 0.017. For all brands, all correlations for depression and major depression retain their significance for this adjusted value, while the aggregate correlations per active compound retain their significance for major depression (Table 3).

**Table 3:** Correlations between year of FDA approval and weighted average rating the aggregates of all drugs sharing the same active compound with more than the indicated number of samples across the online databases in this study, using the date of earliest FDA approval for that active compound (not the individual FDA approvals for brands using that compound which were approved at a later date)

Number of samples required for inclusion	Depression		Major depression		All indications	
	Pearson's *r*	*P*‐value	Pearson's *r*	*P*‐value	Pearson's *r*	*P*‐value
≥1	−0.19	0.1802	−0.24	0.1394	−0.43	**0.0015**
≥5	−0.28	0.0599	−0.77	**<0.0001**	−0.43	**0.0015**
≥10	−0.27	0.0898	−0.70	**0.0002**	−0.43	**0.0017**
≥20	−0.30	0.0857	−0.70	**0.0004**	−0.41	**0.0044**
≥40	−0.40	**0.0279**	−0.77	**0.0002**	−0.39	**0.0090**

Shown are the Pearson correlation coefficient r (denoting the strength of the correlation between the rating and the year of FDA approval of a drug), and the associated *P*‐value. The Bonferroni‐adjusted *P*‐value for significance is P* = 0.017. Aggregate rankings for each active compound: correlation between year of FDA approval and weighted average rating. Values smaller than the (unadjusted) P‐value of 0.05 are denoted in bold.

When calculating Spearman's *ϱ*, the same relationships are maintained.

## Discussion

A large class of explanations for the observed relations stem from economic considerations. Older drugs have long lost their patent protection, and with it part of their earning potential. Former patent holders may either not exist as independent corporate entities any longer, or may have moved on to newer, patent‐protected drugs. There are more market incentives on allocating promotional efforts and marketing budget toward higher‐yield, patent‐protected drugs. The interdependence between which drugs are prescribed and which drugs are marketed is well documented (O'Donoghue et al. 1982; Gupta et al. 2010; Narendran and Narendranathan 2013; Lahey 2014).

Looking into large‐scale patient‐generated databases is, then, of scientific importance for two major reasons: For one, certifying that all viable candidate medications, irrespective of their patent status and marketing expenditures, are taken into consideration in order to render optimal patient treatment decisions. Also, offering generic and potentially equivalent medications the use of which has lapsed because it left the public eye may have a large economic impact: increasing competitiveness via more treatment options holds the promise of decreasing healthcare costs without compromising healthcare quality. Patient approval ratings may offer a good basis for identifying drug candidates to be reevaluated without committing to expensive prospective trials.

Individual selection biases (per database) can be considered negligible because the observed relationships carry across databases, however, systemic selection or general reporting bias might contribute to the results of this study. Hypothetically older patients could receive older drugs stemming from different prescription practices at the time of diagnosis, and are less prone to providing online ratings compared to younger patients who get newer drugs and who may be more prone to using online databases for feedback. This feedback could then be skewed toward one end of the spectrum (e.g., if negative experiences lead to a higher propensity for entering feedback) simply as a consequence of different age bracket representation. This challenge to the study's external validity can be alleviated by correlating with comparative trials which offer point‐wise verification for selected data points. Looking at the observations done in Cipriani et al. (2009); Tedeschini et al. (2011); and Magni et al. (2013), and in particular Anderson (2000), the ranking as provided by the online databases seems to be intact. Even though this proves concordance with clinical trials for only part of the online feedback, the good agreement is encouraging and points to an actual external cause of the observed correlations, rather than to an artifact of data gathering, or a bias inherent only in the online databases.

If indeed younger patients are more critical, for example, because of generally higher expectations regarding the capabilities of medicine or hypothetically because of shifting cultural norms, and if that did explain the relationships observed in this study, a deeper knowledge regarding such an age‐specific negative selection bias could be necessary in order to correctly evaluate newer drugs, specifically in comparison to older drugs. In terms of cost efficiency and immediate feasibility, the best source of large datasets in order to form a more thorough sense of such a bias may also be similar large‐scale user‐driven online databases and their evaluation compared to, for example, the initial phase III studies done in the past, specifically using age‐matched comparisons between then and now.

While the dimensionality of the data as it is currently available does not yet allow for detailed subgroup analyses, both biases and preferences exhibited by different segments of reviewing patients may be heterogeneous not only regarding different age groups but also for characteristics such as treatment resistance. Patients who are in general nonresponders to antidepressants and who eventually move to other, nonmedication‐based modes of therapy may first follow a trace of different antidepressants, all of which would remain ineffectual in that scenario. Depending on such a succession of drugs starting with a comparatively older or newer agent, one patient suffering from therapy‐resistant depression may lead to many more reviews compared to a therapy responder, and thus, as a kind of emergent superreviewer, contribute to the observed correlations if the succession of drugs were to be distributed unequally between older and newer agents.

Nonresponders who try various drugs in search of an effective treatment could change the correlations observed in this study in three ways. If the succession of drugs tried by nonresponders was overall distributed equally between older and newer agents that would weaken any correlation but not change its slope, that is, the direction of the correlation. If, however, a majority of nonresponders could be assumed to start with more recent drugs before moving on to older antidepressants, then that would at first lead to a preponderance of low ratings for newer drugs, which could positively contribute to the correlations as found in this study and explain a portion of the effect magnitude.

Conversely, if nonresponders were on average assumed to start out with older antidepressants, rating them as low satisfaction while gradually moving onto newer drugs as they emerged, such a sequence of reviews would counteract the correlations as observed in this study, which would point at the actual causal factors being of sufficient import to compensate.

However, given the overall effect size, the causality being in essence dominated by a single subgroup seems unlikely, although subgroups divided along various axes could well contribute different portions to the overall correlation.

A preliminary observation to be looked into as a follow‐up is that patient satisfaction does not seem to predict doctor satisfaction with a drug (as given by the RateRx data), a finding especially relevant given the oversize impact of the drug recommendations and information given by healthcare providers (Larkin et al. 2015).

From a methodological standpoint it can be concluded that the exploitation of online databases enables a big data analysis approach which potentially offers a new tool for optimizing healthcare cost and for screening and selecting potential clinical trials and clinical comparative studies for their expected impact before conducting them.

## Conclusion

Patient‐reported drug reviews have reached a sample size and overall concordance across different online databases sufficient that they may now constitute a valid screening tool to elucidate both new trends in comparative drug performance outside of prospective research studies, and to identify critical objectives for future comparative research. This study points toward a strong relationship between the time since a drug's introduction and higher patient satisfaction for both the self‐reported indications of depression and major depression. This relationship was maintained both for aggregate data across the major user‐populated drug review databases as well as when considering individual trade names. Economic incentives in marketing new drugs with a lower score in patient satisfaction over older drugs without patent protection may constitute a causative factor for that troubling observation.

## Author Contributions

S.S. and R.A. designed the study. S.S. and R.A. did the literature review. S.S, R.A., and P.M. did the data collection. S.S., R.A., and C.C. performed the statistical data analysis. All authors contributed to the interpretation and discussion of the results either at the study analysis or manuscript‐writing phase. This report was mainly written by R.A. with assistance from S.S. and C.C. All authors critically commented on and revised the manuscript.

## Disclosure

All authors have no competing interests to declare.
